# Intermittent Fasting Exacerbates the Acute Immune and Behavioral Sickness Response to the Viral Mimic Poly(I:C) in Mice

**DOI:** 10.3389/fnins.2019.00359

**Published:** 2019-04-17

**Authors:** Geraldine Zenz, Angela Jačan, Florian Reichmann, Aitak Farzi, Peter Holzer

**Affiliations:** ^1^Research Unit of Translational Neurogastroenterology, Division of Pharmacology, Otto Loewi Research Center, Medical University of Graz, Graz, Austria; ^2^CBmed GmbH—Center for Biomarker Research in Medicine, Graz, Austria; ^3^BioTechMed-Graz, Graz, Austria

**Keywords:** intermittent fasting, alternate day fasting, Poly(I:C), sickness response, pro-inflammatory cytokines, hypothalamus, neuropeptide Y, corticosterone

## Abstract

Intermitted fasting and other forms of calorie restriction are increasingly demonstrated to exert potential health benefits. Interestingly, restricted feeding is also able to mitigate sickness in response to bacterial factors stimulating Toll-like receptor 4 (TLR4). However, little is known about how fasting modifies the activity of virus-associated molecular patterns. We therefore analyzed the impact of an intermittent fasting (IF) regimen on the immune and behavioral response to the TLR3 agonist and viral mimic polyinosinic:polycytidylic acid [Poly(I:C)] in mice. The effects of intraperitoneally injected Poly(I:C) (12 mg/kg) on plasma and cerebral cytokine expression and behavior (locomotion, exploration, and ingestion) were examined in male C57BL/6N mice under control conditions and following a 9 days period of intermittent (alternate day) fasting (IF). Poly(I:C) increased the circulating levels of cytokines (TNF-α, MCP-1, IL-6, IL-10, IFN-α, IFN-γ), an effect amplified by IF. In addition, IF aggravated sickness behavior in response to Poly(I:C), while cerebral cytokine expression was enhanced by application of Poly(I:C) in the absence of a significant effect of IF. Furthermore, IF augmented the expression of neuropeptide Y (NPY) mRNA in the hypothalamus and increased the plasma levels of corticosterone, while Poly(I:C) had little effect on these readouts. Our data show that IF does not abate, but exaggerates the immune and sickness response to the viral mimic Poly(I:C). This adverse effect of IF occurs despite increased hypothalamic NPY expression and enhanced plasma corticosterone. We therefore propose that the effects of IF on the immune and behavioral responses to viral and bacterial factors are subject to different neuronal and neuroendocrine control mechanisms.

## Introduction

Stimulation of the peripheral immune system is known to bring along changes in brain function and behavior. One widely studied example in this context is the pathogen-associated molecular pattern (PAMP) lipopolysaccharide (LPS). When applied to the periphery, LPS evokes an immune reaction by binding to Toll-like receptor 4 (TLR4), which is a subgroup member of pattern recognition receptors (PRR) that are part of the innate immune system and contribute to the first-line immune response evoked by invading pathogens (Poltorak et al., 1998; Kawai and Akira, 2011). Alike most PRRs, TLR4 is found on the surface of numerous immune cells, and its stimulation leads to elevated cytokine levels in the plasma of organisms exposed to LPS (Kawai and Akira, 2011). Furthermore, animals injected intraperitoneally (i.p.) with LPS also show enhanced expression of cytokines within the brain and activation of the hypothalamic-pituitary-adrenal (HPA) axis as reflected by elevated circulating corticosterone (CORT) levels (Lenczowski et al., 1997; Farzi et al., 2015b; Mayerhofer et al., 2017). These findings attest to a close immune synergy of the periphery and brain. While some of the changes observed in the brain are adaptive physiological processes that help the individual to overcome infection, in humans elevated cytokine levels as well as circulating LPS were found to correlate with symptoms of depression and anxiety (Raison et al., 2006; Vogelzangs et al., 2016).

There is increasing interest in how to interfere with undesired mirroring processes along the immune-brain-axis and to restrict the pro-inflammatory state within the periphery and brain. One novel approach is to subject rodents that are challenged by bacterial infection or LPS to a fasting regimen (Matsuzaki et al., 2001; Radler et al., 2014; Godinez-Victoria et al., 2014). It has been reported that a 50% calorie restriction suppresses sickness behavior in mice treated with LPS and increases hypothalamic neuropeptide Y (NPY) mRNA expression, which has been indirectly correlated with a reduction of microglial activation (MacDonald et al., 2011; Radler et al., 2015). While these reports attest to beneficial effects of calorie restriction on the adverse effects of TLR4 stimulation by bacterial PAMPs, little is known about other PAMPs that stimulate distinct PRRs and evoke expression of a different pattern of pro-inflammatory cytokines. Therefore, it was the overall aim of this study to evaluate whether sickness induced by polyinosinic:polycytidylic acid [Poly(I:C)] is mitigated by intermittent fasting (IF), a dietary regimen which allows *ad libitum* (AL) feeding every other day interrupted by 24 h periods without food. Poly(I:C) is a synthetic double-stranded RNA (dsRNA) molecule that activates the innate immune system in a similar manner as viral infection, stimulating TLR3 in certain immune cells, including myeloid dendritic cells and macrophages (Watanabe et al., 2011; Jensen and Thomsen, 2012). TLR3 is present in endosomal compartments, thus Poly(I:C) must be internalized to stimulate an immune response, which is thought to be mediated partly by CD14-dependent and clathrin-mediated mechanisms. Following endocytosis, the synthetic dsRNA molecule binds to Mex3B which promotes the binding of Poly(I:C) to TLR3 and eventually causes downstream signaling, including upregulated expression of interferons (IFN) of type I, tumor necrosis factor-α (TNF-α), and interleukin-6 (IL-6) (Cunningham et al., 2007; Zhu et al., 2016).

The specific objective of the present study was to investigate the influence of a 9 days IF regimen on the effects of a sub-septic dose of i.p. administered Poly(I:C) (12 mg/kg) on cytokine, neuropeptide and neuroendocrine readouts in blood plasma and brain as well as on behavior of C57BL/6N mice. A particular advantage of IF is that mice maintain their overall food intake and body weight when compared to animals with food AL, while they enjoy the same health benefits seen in other fasting interventions like calorie restriction (Gotthardt et al., 2016). First, the time course of the sickness response to Poly(I:C) was explored in a homecage-like environment to determine when the disturbances of locomotor, exploratory and ingestive behavior reach their maximum. The second experiment assessed whether IF might affect exploratory behavior of Poly(I:C) treated mice in the open field (OF) test at the time when sickness climaxes. Furthermore, we measured cytokine levels in blood plasma and pro-inflammatory cytokine and NPY mRNA expression in the hypothalamus. In addition, the activity of the HPA axis was determined by measuring CORT levels in the plasma.

## Materials and Methods

### Experimental Animals

Male C57BL/6N mice (*n* = 45), received from Charles River Laboratories (Sulzfeld, Germany) at the age of 8 weeks, were habituated to the new environment for at least one week before any intervention was undertaken. Tap water and standard laboratory chow were provided AL, unless stated otherwise. The animals were kept in groups of two under a 12 h light/dark cycle (lights on at 6:00 h, lights off at 18:00 h). The room temperature was set at 22°C, and a relative air humidity of 50% was maintained. All housing conditions were tightly controlled.

### Ethics Statement

All experiments and the number of animals used were approved by an ethical committee at the Federal Ministry of Science, Research and Economy of the Republic of Austria (BMWF-66010/0102-WF/V/3b/2017). The procedures were performed according to the Directive of the European Parliament and of the Council of 22 September 2010 (2010/63/EU). Special care was taken and the experiments were designed in such a way that the suffering and the total number of animals used was minimized.

### Reagents and Dosing

The synthetic analog of dsRNA, Poly(I:C), was purchased from Invivogen (Toulouse, France, catalog number: tlrl-picw; free from microbial contaminants; low molecular weight: 0.2–1 kb). Poly(I:C) was dissolved in pyrogen-free sterile saline provided by Invivogen, as suggested by the manufacturer. Poly(I:C) and pyrogen-free sterile saline (0.9% NaCl) were injected i.p. at the same volume (10 μl/g body weight). For the induction of a TLR3 dependent sickness response in mice a dose of 12 mg/kg Poly(I:C) was chosen (Cunningham et al., 2007; Michalovicz and Konat, 2014).

### Behavioral Testing

Mice were habituated to the test room for at least 24 h (lights on at 6:00 h, lights off at 18:00 h, temperature set point 22°C, relative air humidity 50%, maximal light intensity 100 lux).

### LabMaster Test

Locomotion, exploratory behavior as well as water and food intake of the test mice (aged 9–10 weeks) were continuously recorded in the homecage-like environment of the LabMaster system (TSE Systems, Bad Homburg, Germany), as described previously (Painsipp et al., 2013). In short, transparent LabMaster test cages (type III, 42.0 × 26.5 × 15.0 cm, length × width × height) were surrounded by two frames sending out infrared beams to measure vertical exploratory behavior (rearing) as well as horizontal locomotor activity by counts of infrared beam interruptions. The lower infrared frame was positioned 2.0 cm above the cage floor, while the upper frame was positioned at a distance of 4.3 cm above the lower one. Furthermore, two weight transducers attached to the cage lids were used to evaluate ingestive behavior, as a feeding bin, filled with standard rodent chow, and a drinking bottle were attached to the transducers throughout the experiment. All recording devices were connected to a personal computer which was used to collect and analyze the data with the LabMaster software. Food and water intake was expressed in grams of food ingested per body weight (g/g) and milliliter of water ingested per body weight (ml/g), respectively. The animals were conditioned to the drinking bottles used in the LabMaster system and to single housing for at least 72 h before placing them in the test cages. Another habituation period of at least 24 h enabled the mice to get used to the LabMaster cages before the experiment was started. Within the LabMaster system, mice were kept one by one in order to enable accurate activity measurements of each mouse. All LabMaster parameters were recorded for 12 h after the injection of either saline or Poly(I:C). Injections were performed approximately 1 h before the dark-cycle began, at 17:00 h.

### Open Field (OF) Test

The OF box (50 × 50 × 30 cm, length × width × height; opaque gray plastic) was illuminated by 35 lux at floor level (Painsipp et al., 2013). Mice (aged 11 weeks at the time of the OF test) were placed individually into one corner of the OF and their behavior was recorded and tracked by a video camera above the center of the OF (VideoMot2 software; TSE Systems). The OF box ground area was divided into a 36 × 36 cm central area, surrounded by the outer border zone, and mice were allowed to explore the new environment for 5 min. After each trial, the OF was cleaned with 70% ethanol, followed by water.

### Experimental Protocols

**FIGURE 1:**
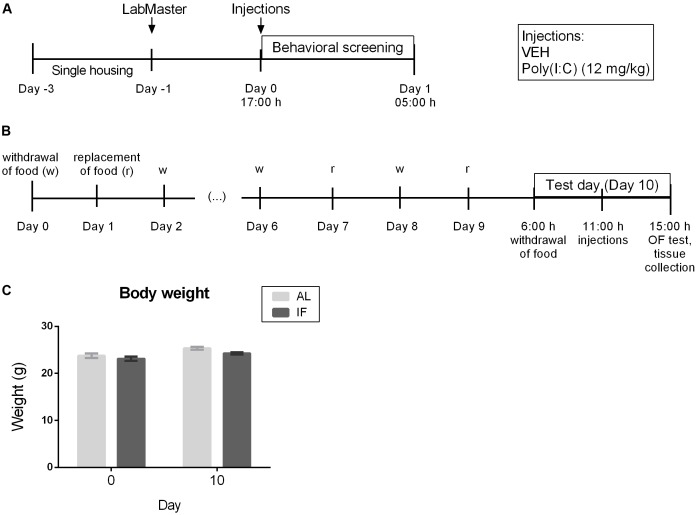
Experimental time lines and body weight recordings. **(A)** Time course of the effects of i.p. injection of vehicle or Poly(I:C) (12 mg/kg) on locomotor and exploratory behavior as well as food and water intake assessed in the LabMaster system (*n* = 6). **(B)** Effect of intermittent fasting (IF) for 9 days on the sickness behavior in the open field (OF) evoked by i.p. injection of vehicle or Poly(I:C) (12 mg/kg) on day 10 (*n* = 7–9). **(C)** Body weight of mice at days 0 and 10 (*n* = 6). RM two-way ANOVA results: *F*(1, 10) = 0.7946, *p* = 0.3937 for interaction of time/feeding in body weight; *F*(1, 10) = 25.84, *p* ≤ 0.001 for main effect of time on body weight; *F*(1, 10) = 3.199, *p* = 0.1040 for main effect of feeding regimen on body weight. Abbreviations: AL, *ad libitum* feeding.

**FIGURE 2:**
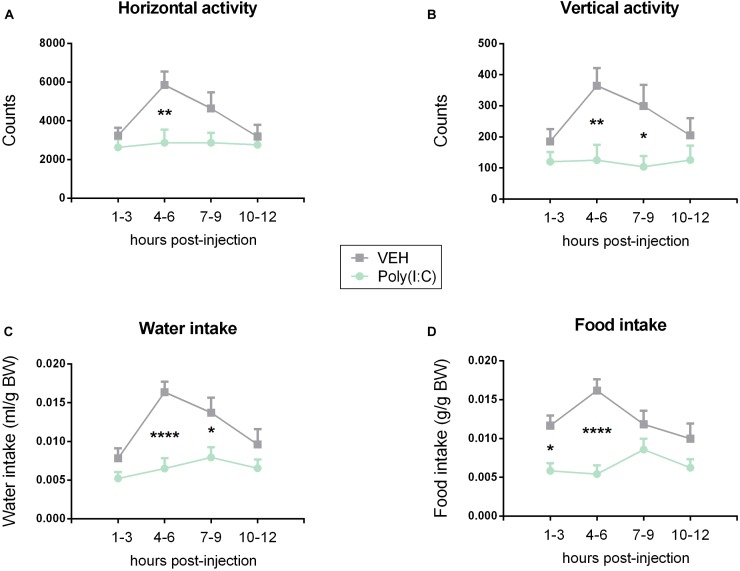
Effect of Poly(I:C) on locomotion (horizontal activity) **(A)**, exploration (vertical activity, rearing) **(B)**, water **(C)**, and food **(D)** intake. Poly(I:C) (12 mg/kg) or vehicle (VEH) was injected i.p. at 0 h, and the test parameters were recorded at the subsequent time intervals shown. Values represent means + SEM, *n* = 6. Results of RM two-way ANOVA: *F*(3, 102) = 2.559, *p* = 0.0592 for interaction of time/treatment in locomotion; *F*(3, 102) = 3.400, *p* ≤ 0.05 for main effect of time on locomotion; *F*(1, 34) = 6.412, *p* ≤ 0.05 for main effect of i.p. treatment on locomotion; *F*(3, 102) = 1.992, *p* = 0.1199 for interaction of time/treatment in exploration; *F*(3, 102) = 1.855, *p* = 0.142 for main effect of time on exploration; *F*(1, 34) = 10.31, *p* ≤ 0.01 for main effect of i.p. treatment on exploration; *F*(3, 102) = 2.661, *p* = 0.0521 for interaction of time/treatment in water intake; *F*(3, 102) = 5.135, *p* ≤ 0.01 for main effect of time on water intake; *F*(1, 34) = 27.12, *p* ≤ 0.0001 for main effect of i.p. treatment on water intake; *F*(3, 102) = 3.256, *p* ≤ 0.05 for interaction of time/treatment in food intake; *F*(3, 102) = 1.730, *p* = 0.1656 for main effect of time on food intake; *F*(1, 34) = 26.87, *p* ≤ 0.0001 for main effect of i.p. treatment on food intake. Results of Bonferroni’s multiple comparison *post hoc* test: ^∗^*p* ≤ 0.05, ^∗∗^*p* ≤ 0.01, ^∗∗∗∗^*p* ≤ 0.0001. Abbreviations: BW, body weight.

Two experiments were performed (Figure 1). The first experiment (Figure 1A, 2) evaluated the effects of i.p. injected Poly(I:C) (12 mg/kg) on locomotion, exploration, feeding and drinking behavior in the LabMaster system in order to determine when the sickness response peaks following i.p. injection. Food and water were available AL during the testing period of experiment 1.

The second experiment (Figure 1B,C, 3–6) was carried out to evaluate whether IF for 9 days influences the immune and behavioral response to i.p. Poly(I:C) injection on day 10 which for the IF group was a fasting day. Mice were weighed at the beginning of the protocol on days 0 and 10 at 6:00 h before i.p. treatment in order to analyze the effect of the feeding regimens on the body weight. On the test day (day 10), mice of the IF group were deprived of food at 6:00 h (beginning of the light phase), 5 h before injection of Poly(I:C) or vehicle, a schedule that was similar to a protocol used to test LPS (MacDonald et al., 2011). Food was also withheld during the following treatment and test period, whereas control animals had AL access to food. Beginning at 11:00 h, mice were each injected in intervals of 10 min to ensure Poly(I:C) and vehicle treatment exactly 4 h prior to the behavioral test. The sickness behavior in experiment 2 was assessed with the OF test. Directly after the OF test, the animals were sacrificed to collect plasma and brain for molecular analysis.

**FIGURE 3:**
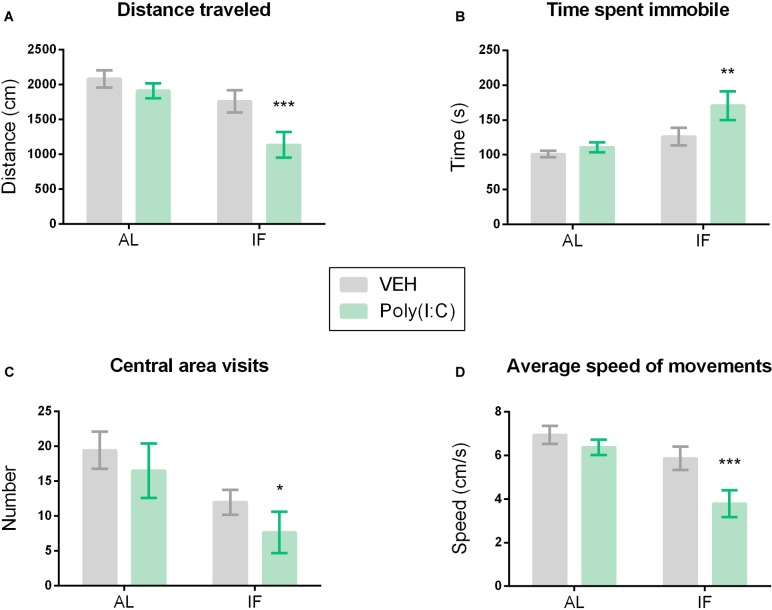
Intermittent fasting induces sickness behavior in response to Poly(I:C). Mice were kept on an *ad libitum* feeding regimen (AL) or deprived of food on every other day (IF) for 9 days. On day 10, mice were injected i.p. with vehicle (VEH) or Poly(I:C) (12 mg/kg) and 4 h later subjected to the open field (OF) test to evaluate sickness behavior: **(A)** distance traveled, **(B)** time spent immobile, **(C)** visits to the central area of the OF, **(D)** average speed of movements. Values represent means ± SEM, *n* = 7–9. Results of two-way ANOVA: *F*(1, 29) = 2.164, *p* = 0.1521 for interaction of feeding regimen/Poly(I:C) in distance traveled; *F*(1, 29) = 12.73, *p* ≤ 0.01 for main effect of feeding regimen on distance traveled; *F*(1, 29) = 6.674, *p* ≤ 0.05 for main effect of Poly(I:C) on distance traveled; *F*(1, 29) = 1.575, *p* = 0.2195 for interaction of feeding regimen/Poly(I:C) in time spent immobile; *F*(1, 29) = 9.330, *p* ≤ 0.01 for main effect of feeding regimen on time spent immobile; *F*(1, 29) = 3.813, *p* = 0.0606 for main effect of i.p. treatment on time spent immobile; *F*(1, 29) = 0.05753, *p* = 0.8121 for interaction of feeding/Poly(I:C) in number of central area visits; *F*(1, 29) = 7.710, *p* ≤ 0.01 for main effect of IF on number of central area visits; *F*(1, 29) = 1.537, *p* = 0.2249 for main effect of i.p. treatment on number of central area visits; *F*(1, 29) = 2.163, *p* = 0.1521 for interaction of feeding regimen/Poly(I:C) in speed of movements; *F*(1, 29) = 12.72, *p* ≤ 0.01 for main effect of feeding regimen on speed of movements; *F*(1, 29) = 6.673, *p* ≤ 0.05 for main effect of Poly(I:C) on speed of movements. Results of Sidak’s multiple comparison test: ^∗^*p* ≤ 0.05, ^∗∗^*p* ≤ 0.01, ^∗∗∗^*p* ≤ 0.001 compared to AL/VEH.

**FIGURE 4:**
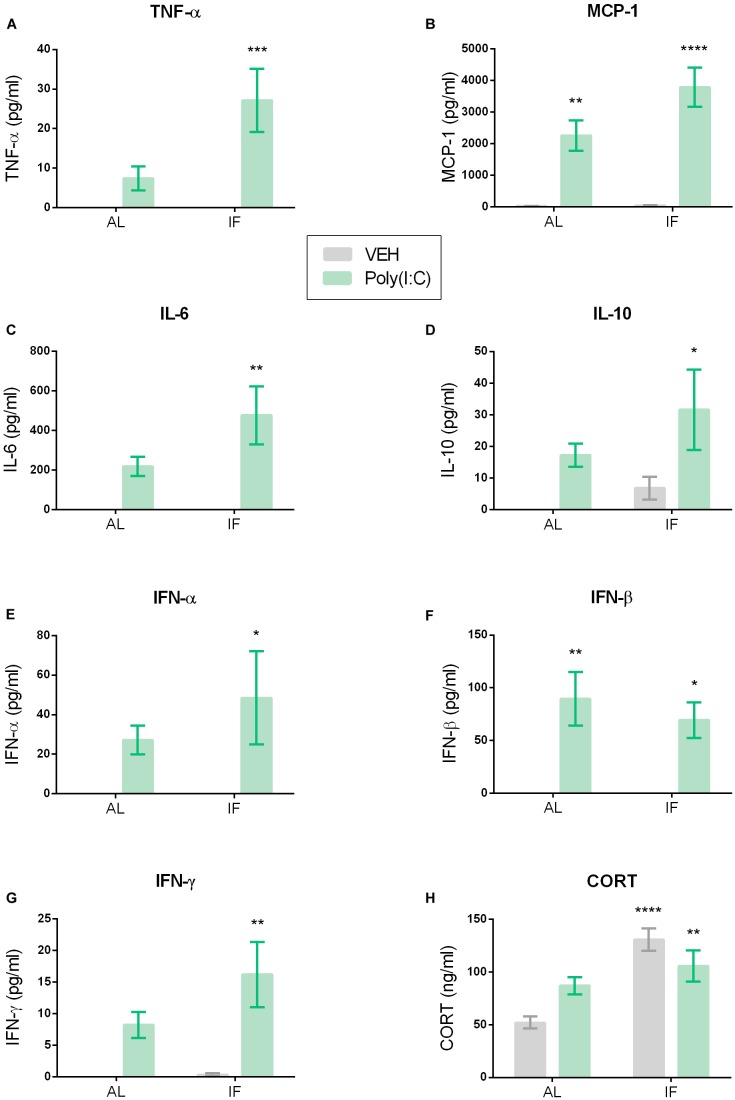
Intermittent fasting impacts Poly(I:C)-induced changes in plasma cytokine and corticosterone (CORT) levels. Mice were kept on an *ad libitum* feeding regimen (AL) or deprived of food on every other day (IF) for 9 days. On day 10, mice were injected i.p. with vehicle (VEH) or Poly(I:C) (12 mg/kg) and 4 h later plasma samples were drawn to evaluate circulating cytokine and CORT levels. Values represent means ± SEM, **(A,B,E,F)** *n* = 7–8, **(C,D,G)** *n* = 6–8, **(H)** *n* = 7–9. Results of two-way ANOVA: *F*(1, 26) = 4.636, *p* ≤ 0.05 for interaction of feeding regimen/Poly(I:C) in plasma TNF-α; *F*(1, 26) = 4.636, *p* ≤ 0.05 for main effect of feeding regimen on plasma TNF-α; *F*(1, 26) = 14.24, *p* ≤ 0.001 for main effect of i.p. treatment on plasma TNF-α levels; *F*(1, 26) = 3.222, *p* = 0.0843 for interaction of feeding regimen/Poly(I:C) in plasma MCP-1; *F*(1, 26) = 3.387, *p* = 0.0772 for main effect of feeding regimen on plasma MCP-1; *F*(1, 26) = 50.54, *p* ≤ 0.0001 for main effect of Poly(I:C) on plasma MCP-1; *F*(1, 24) = 2.062, *p* = 0.1639 for interaction of feeding regimen/Poly(I:C) in plasma IL-6; *F*(1, 24) = 2.062, *p* = 0.1639 for main effect of feeding on plasma IL-6; *F*(1, 24) = 14.98, *p* ≤ 0.001 for main effect of Poly(I:C) on plasma IL-6; *F*(1, 25) = 0.2464, *p* = 0.6240 for interaction of feeding regimen/Poly(I:C) in plasma IL-10; *F*(1, 25) = 1.936, *p* = 0.1764 for main effect of feeding on plasma IL-10; *F*(1, 25) = 7.620, *p* ≤ 0.05 for main effect of Poly(I:C) on plasma IL-10; *F*(1, 26) = 0.6399, *p* = 0.4310 for interaction of feeding regimen/Poly(I:C) in plasma IFN-α; *F*(1, 26) = 0.6399, *p* = 0.4310 for main effect of feeding on plasma IFN-α; *F*(1, 26) = 8.164, *p* ≤ 0.01 for main effect of Poly(I:C) on plasma IFN-α; *F*(1, 25) = 0.3809, *p* = 0.5427 for interaction of feeding regimen/Poly(I:C) in plasma IFN-β; *F*(1, 25) = 0.3809, *p* = 0.5427 for main effect of feeding on plasma IFN-β; *F*(1, 25) = 23.79, *p* ≤ 0.0001 for main effect of Poly(I:C) on plasma IFN-β; *F*(1, 25) = 1.492, *p* = 0.2334 for interaction of feeding regimen/Poly(I:C) in plasma IFN-γ; *F*(1, 25) = 1.787, *p* = 0.1933 for main effect of feeding on plasma IFN-γ; *F*(1, 25) = 14.90, *p* ≤ 0.001 for main effect of Poly(I:C) on plasma IFN-γ; *F*(1, 27) = 7.638, *p* ≤ 0.05 for interaction of feeding regimen/Poly(I:C) in plasma CORT; *F*(1, 27) = 20.14, *p* ≤ 0.0001 for main effect of feeding on plasma CORT; *F*(1, 27) = 0.2125, *p* = 0.6485 for main effect of i.p. treatment on plasma CORT. Results of Sidak’s multiple comparison test: ^∗^*p* ≤ 0.05, ^∗∗^*p* ≤ 0.01, ^∗∗∗^*p* ≤ 0.001, ^∗∗∗∗^*p* ≤ 0.0001 compared to AL/VEH.

**FIGURE 5:**
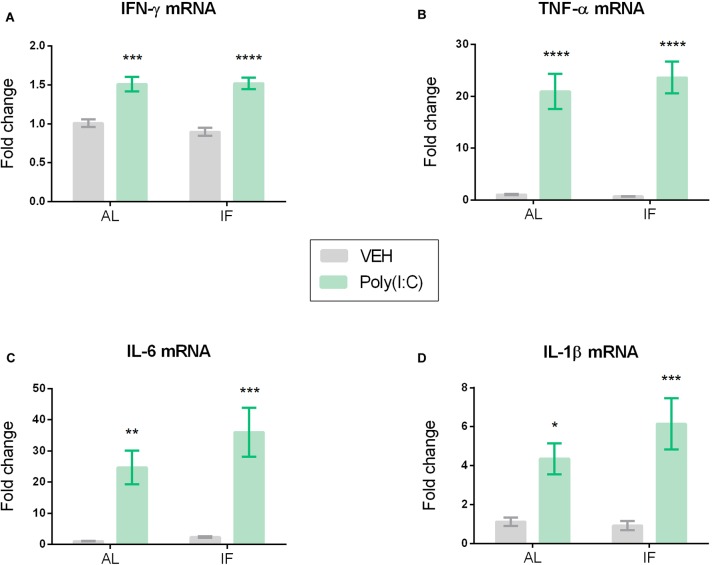
Hypothalamic cytokine mRNA expression of **(A)** IFN-γ, **(B)** TNF-α, **(C)** IL-6, and **(D)** IL-1β is elevated by peripheral Poly(I:C). Mice were kept on an *ad libitum* feeding regimen (AL) or deprived of food on every other day (IF) for 9 days. On day 10, mice were injected i.p. with vehicle (VEH) or Poly(I:C) (12 mg/kg) and 4 h later brains were extracted to evaluate hypothalamic cytokine mRNA expression. Values represent means ± SEM, **(A–D)** *n* = 7–8. Results of two-way ANOVA: *F*(1, 26) = 0.6824, *p* = 0.4163 for interaction of feeding regimen/Poly(I:C) in IFN-γ mRNA; *F*(1, 26) = 0.4720, *p* = 0.4982 for main effect of feeding on IFN-γ mRNA; *F*(1, 26) = 60.30, *p* ≤ 0.0001 for main effect of Poly(I:C) on IFN-γ mRNA; *F*(1, 25) = 0.3376, *p* = 0.5664 for interaction of feeding regimen/Poly(I:C) in TNF-α mRNA; *F*(1, 25) = 0.2003, *p* = 0.6583 for main effect of feeding on TNF-α mRNA; *F*(1, 25) = 70.09, *p* ≤ 0.0001 for main effect of Poly(I:C) on TNF-α mRNA; *F*(1, 26) = 0.9556, *p* = 0.3373 for interaction of feeding regimen/Poly(I:C) in IL-6 mRNA; *F*(1, 26) = 1.510, *p* = 0.2301 for main effect of feeding on IL-6 mRNA; *F*(1, 26) = 31.27, *p* ≤ 0.0001 for main effect of Poly(I:C) on IL-6 mRNA; *F*(1, 26) = 1.401, *p* = 0.2473 for interaction of feeding regimen/Poly(I:C) in IL-1β mRNA; *F*(1, 26) = 0.8909, *p* = 0.3539 for main effect of feeding on IL-1β mRNA; *F*(1, 26) = 25.39, *p* ≤ 0.0001 for main effect of Poly(I:C) on IL-1β mRNA. Results of Sidak’s multiple comparison test: ^∗^*p* ≤ 0.05, ^∗∗^*p* ≤ 0.01, ^∗∗∗^*p* ≤ 0.001, ^∗∗∗∗^*p* ≤ 0.0001 compared to AL/VEH.

**FIGURE 6:**
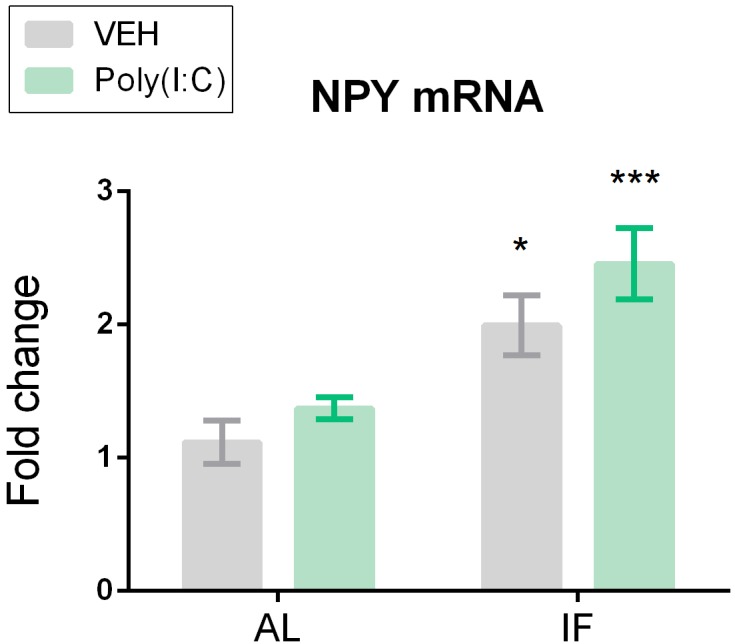
Neuropeptide Y (NPY) mRNA expression is significantly upregulated in the hypothalamus of intermittently fasted mice. Mice were kept on an *ad libitum* feeding regimen (AL) or deprived of food on every other day (IF) for 9 days. On day 10, mice were injected i.p. with vehicle (VEH) or Poly(I:C) (12 mg/kg) and 4 h later brains were extracted to evaluate NPY mRNA expression in the hypothalamus. Values represent means ± SEM, *n* = 6–8. Results of two-way ANOVA: *F*(1, 25) = 0.2708, *p* = 0.6074 for interaction of feeding regimen/Poly(I:C) in NPY mRNA;*F*(1, 25) = 24.45, *p* ≤ 0.0001 for main effect of feeding regimen on NPY mRNA; *F*(1, 25) = 3.262, *p* = 0.083 for main effect of Poly(I:C) on NPY mRNA. Results of Sidak’s multiple comparison test: ^∗^*p* ≤ 0.05, ^∗∗∗^*p* ≤ 0.001 compared to AL/VEH.

### Blood Sampling and Brain Tissue Harvesting

After the mice had been deeply anesthetized with i.p. pentobarbital (150 mg/kg), blood was drawn by cardiac puncture with a syringe that was filled with 100 μl of sodium citrate (3.8%) as an anticoagulant. The sampled blood was centrifuged for 15 min at 1000 × *g* and 4°C to collect supernatant plasma which was stored at -70°C until use. Brains were collected immediately after blood sampling and instantly frozen in 2-methylbutane (Sigma-Aldrich, Vienna, Austria) on dry ice for 10 s. Subsequently, brains were kept at -70°C until microdissection.

### Brain Microdissection

Microdissections were performed on a cold plate (Weinkauf Medizintechnik, Forchheim, Germany) set at -20°C as previously described (Brunner et al., 2014). Care was taken to thoroughly clean the working area and dissection instruments with RNase AWAY (Carl Roth, Karlsruhe, Germany) before each dissection. Hypothalamic brain areas (Bregma +0.38 to -2.92) were collected in MagnaLyser bead tubes (catalog number: 03358 941 001, Roche Diagnostics, Rotkreuz, Switzerland) filled with Precellys beads (Peqlab, Erlangen, Germany) and stored at -70°C until RNA extraction.

### Circulating Cytokines and Corticosterone

Cytokine concentrations in plasma samples were evaluated using ProcartaPlex^TM^ immunoassays (eBioscience, San Diego, CA, United States) according to the manufacturer’s specifications (Mayerhofer et al., 2017). Fluorescent signals were quantified with the Bio-Plex 200 multiplex suspension array system equipped with Luminex^®^ xMAP^®^ technology combined with the Bio-Plex 5.0 software (BioRad, Hercules, CA, United States).

Cytokines that were too low to be detected were excluded from further analysis. All cytokine concentrations were evaluated in duplicates. It should not go unmentioned at this point that IL-1β levels were analyzed in the blood plasma, but were too low to be detected throughout all groups and will not be displayed in the results part.

Corticosterone plasma levels were determined with a specific enzyme immunoassay kit (Assay Designs, Ann Arbor, MI, United States) with a sensitivity of 0.027 ng/ml as previously described (Farzi et al., 2015b) and according to the manufacturer’s specifications.

### RNA Extraction, Reverse Transcription, and Quantitative Real-Time PCR (qPCR) of Brain Tissues

RNA extractions, reverse transcription and qPCR were performed according to the manufacturer’s specifications and described by Fröhlich et al. (2016). Hypothalamic tissue samples were homogenized with the MagnaLyser homogenizer (Roche Diagnostics). RNA was extracted using the RNeasy lipid tissue mini kit (Qiagen, Hilden, Germany). Subsequently, RNA concentrations were determined using NanoDrop (Thermo Fisher Scientific, Vienna, Austria), and 2 μg of RNA of each sample was reverse-transcribed in the Mastercycler Gradient (Eppendorf, Hamburg, Germany), using the high capacity cDNA reverse transcription kit (Thermo Fisher Scientific). Afterward, mRNA levels were quantified in triplicates via qPCR using a LightCycler 480^®^ system with TaqMan gene expression assays for IFN-γ (Mm01168134_m1), IL-1β (Mm00434228_m1), IL-6 (Mm00446190_m1), NPY (Mm03048253_m1), and TNF-α (Mm00443258_m1) and with the TaqMan gene expression master mix (Thermo Fisher Scientific). Negative controls without reverse transcriptase added were included for each treatment group. As reference genes (endogenous housekeeping), PPIL3 (Mm00510343_m1), and ACTB (Mm00607939_s1) were used. In order to quantitate target gene levels relative to controls the 2^-ΔΔCt^ method was used, where the mean value of the vehicle treated group was used as calibrator and group differences were expressed as fold changes.

### Statistics

Statistical analysis of the results was performed using GraphPad^®^ Prism5 (GraphPad Software Inc., La Jolla, CA, United States). In order to analyze the effect of the feeding regimens on body weight and the results of the LabMaster test, repeated measures (RM) two-way analysis of variance (ANOVA) with *post hoc* Bonferroni’s multiple comparison test for each time point was performed. Probability values of *p* ≤ 0.05 were regarded as statistically significant.

The results of the IF experiment were evaluated with two-way ANOVA. In addition, Sidak’s multiple comparison test was used for planned comparison between all treatment groups and the control group with AL access to food injected with vehicle. Extreme outliers, referring to values more than three times the interquartile range off a quartile, were excluded. This correction was applied in the evaluation of circulating cytokines, namely IL-6 (one extreme outlier in each vehicle treated group), IFN-β [one extreme outlier in the IF group injected with Poly(I:C)], IL-10 (one extreme outlier in the AL group injected with vehicle) and IFN-γ (one extreme outlier in the AL group treated with vehicle). Furthermore, one extreme outlier was excluded from the evaluation of CORT levels [IF group treated with Poly(I:C)] and one extreme value was excluded from the analysis of both NPY and TNF-α mRNA expression in brain tissue (IF group injected with vehicle).

## Results

### Sickness Response to i.p. Poly(I:C) Peaks Between 4 and 6 h After Injection

In order to characterize the sickness response to Poly(I:C), mice were screened for behavioral changes in a homecage-like environment. Mice injected with Poly(I:C) differed from vehicle-treated controls in all four parameters under study (horizontal locomotor activity, vertical exploratory activity, water and food intake) as analyzed by RM two-way ANOVA and shown in Figure 2. *Post hoc* Bonferroni’s multiple comparison test revealed a significant attenuation of locomotor, exploratory and ingestive behavior in animals injected with Poly(I:C). The most significant difference between vehicle- and Poly(I:C)-injected animals was seen during the interval 4–6 h post-injection (Figure 2A–D). Food intake, however, was significantly (*p* ≤ 0.05) reduced already within the first 3 h following injection of Poly(I:C). Exploratory activity and water intake were still blunted 7–9 h after treatment of mice with the viral mimic when compared to vehicle-injected controls (*p* ≤ 0.05).

In view of the sickness response being maximal 4–6 h post-treatment in the present experiments as well as published work in which the sickness response peaked 3–4 h following Poly(I:C) administration (Cunningham et al., 2007; Michalovicz and Konat, 2014; Murray et al., 2015), further analyses of Poly(I:C)-evoked immune stimulation and behavioral perturbations were conducted 4 h following injection.

### IF Induces Sickness in Response to Poly(I:C) in the OF Test

The interaction of IF with Poly(I:C)-induced sickness was analyzed in the OF. The readouts of this test reflect behavior in a new environment, which is known to motivate exploration. As shown in Figure 3, the exploratory behavior in the OF was inhibited in mice subjected to both IF and immune stimulation by Poly(I:C). Specifically, the distance traveled, the speed of movements, the number of central area visits and the time spent immobile were altered when IF was followed by Poly(I:C) challenge (Figure 3A–D). Planned comparison following two-way ANOVA (Figure 3) revealed that i.p. injection of Poly(I:C) in combination with AL feeding did not significantly alter behavior in the OF when compared to vehicle-treated control mice under the same feeding regimen (Figure 3A–D). Administration of Poly(I:C) to mice on preceding IF, however, significantly shortened the distance traveled (Figure 3A), significantly prolonged the time spent immobile (Figure 3B) and significantly reduced the number of central area visits (Figure 3C) and the speed of movements (Figure 3D) as compared to AL fed mice injected with vehicle.

It is important to note that IF did not change the body weight of mice when compared to the AL feeding regimen as evaluated by RM two-way ANOVA (Figure 1C). A similar observation was reported by others (Gotthardt et al., 2016).

### IF Amplifies Poly(I:C)-Induced Elevation of Plasma Cytokine Levels

Relative to vehicle, i.p. injection of Poly(I:C) increased the circulating levels of pro- and anti-inflammatory cytokines such as TNF-α, MCP-1, IL-6, IL-10, IFN-α, IFN-β, and IFN-γ when measured 4 h after injection (Figure 4A–G). Planned comparison following two-way ANOVA (Figure 4) revealed that, in most cases, IF further enhanced this effect. Specific analysis disclosed that the plasma levels of TNF-α, IL-6, IL-10, IFN-α, and IFN-γ were significantly higher in the IF/Poly(I:C) group compared with the AL/vehicle group, but were not significantly increased in the AL/Poly(I:C) group (Figure 4A,C–E,G). In contrast, the circulating levels of MCP-1 and IFN-β were significantly enhanced in both the AL/Poly(I:C) and IF/Poly(I:C) group relative to the AL/vehicle group (Figure 4B,F).

### Plasma CORT Levels Are Raised in Intermittently Fasted Mice

The circulating CORT levels were significantly different between the treatment groups as revealed by two-way ANOVA (Figure 4H). Planned comparison showed that it was IF which enhanced plasma CORT, because the CORT levels in both the IF/vehicle and IF/Poly(I:C) group were significantly higher than in the AL/vehicle group (Figure 4H).

### Poly(I:C) Induces Cytokine mRNA Expression in the Hypothalamus

Poly(I:C) altered the expression of pro-inflammatory cytokine mRNA in the hypothalamus of mice in both feeding regimens (Figure 5). Planned comparison following two-way ANOVA disclosed that the levels of IFN-γ mRNA, TNF-α mRNA, IL-6 mRNA, and IL-1β mRNA in the hypothalamus were significantly elevated by Poly(I:C) independently of feeding regimen (Figure 5A–D).

### IF Enhances Expression of NPY mRNA in the Hypothalamus

Hypothalamic expression levels of NPY mRNA differed between the treatment groups under study as shown by two-way ANOVA. Planned comparison revealed that it was the IF regimen which affected the hypothalamic expression of NPY as the NPY mRNA levels were upregulated both in the IF/VEH group and IF/Poly(I:C) group (Figure 6).

## Discussion

There is evidence that feeding restriction has beneficial effects on several health issues including some types of cancer and autoimmune disease such as multiple sclerosis, which is related to stimulation of repair and replacement mechanisms in response to the metabolic intervention (Lee et al., 2012; Lv et al., 2014; Choi et al., 2016, 2017). However, while some studies suggest that a restriction of calories might also ameliorate the sickness due to TLR4 stimulation by bacterial LPS (Inoue and Luheshi, 2010; MacDonald et al., 2011), this is the first study to assess the impact of IF on the peripheral and cerebral immune responses and behavioral manifestations due to TLR3 stimulation by the viral mimic Poly(I:C). We chose IF as the feeding intervention for the current study as it represents a milder form of fasting compared to calorie restriction (Gotthardt et al., 2016). In this context it should be noted that rodents under calorie restriction are usually provided once daily with food containing a certain amount of calories and will additionally undergo a period of IF after finishing their meal, which exacerbates the fasting effect. In the present study the animals were subjected to a strict IF scheme for 9 days, and the interaction between IF and the Poly(I:C)-induced immune and sickness response was evaluated on the following day after withdrawal of food.

### IF Exaggerates the Behavioral Sickness Response to Poly(I:C)

The current data show that activation of the TLR3 pathway by i.p. administration of the dsRNA mimic Poly(I:C) (12 mg/kg) leads to a behavioral sickness response in the homecage-like environment of the LabMaster system. The signs of sickness observed in this system included a decrease in locomotor activity and exploratory rearing behavior, as well as a diminution of water and food intake. Taking all measurements into account, a clear sickness response was evident around 4–6 h post-injection, which is in line with several other reports that the same dose of Poly(I:C) causes a peak of sickness about 4 h following treatment (Cunningham et al., 2007; Michalovicz and Konat, 2014; Weintraub et al., 2014). For that reason, the impact of IF on the molecular and behavioral response to Poly(I:C) was further analyzed at this time point.

In contrast to the observations in the homecage-like LabMaster setting, i.p. Poly(I:C) alone failed to significantly alter exploratory behavior in the OF test 4 h post-injection. This is in line with previous reports showing stronger sickness behavior in the LabMaster compared to the OF (Farzi et al., 2015b). The OF test involves exposure of the animals to a novel environment and in this aspect differs markedly from the LabMaster setting in which the animals were allowed to habituate for at least 1 day. We therefore suppose that the intrinsic motivation of mice to explore new environments may have overridden the obvious but moderate sickness response that was evident in the LabMaster system. We are affirmed in this conclusion by the observation that under IF conditions Poly(I:C) was able to induce particular signs of sickness behavior in the OF test along with distinct molecular perturbations in blood plasma and brain. At this point, it should be mentioned that IF itself did not notably influence the parameters under study and that mice maintained their overall body weight during IF, which has already been reported by others (Gotthardt et al., 2016).

Unlike the beneficial effect of food restriction on the immune and sickness response to TLR4 stimulation by LPS, various molecular and behavioral effects of TLR3 stimulation by Poly(I:C) were exaggerated by IF. On the basis of this unexpected outcome we propose that the metabolic effects of IF may be disadvantageous for particular neuroimmune processes. It might be the metabolic change that comes along with the withdrawal of food that amplifies the immune reaction and thereby evokes the behavioral changes seen. This is in line with work of Wang et al. (2016) who showed that a comparably high dose of Poly(I:C) (30 mg/kg) injected retro-orbitally followed by i.p. 2-deoxy-D-glucose (2-DG), an inhibitor of glycolysis, was lethal in mice within a 24 h period after treatment, while mice in the control group co-injected with vehicle survived the Poly(I:C) challenge. The adverse effect of Poly(I:C) in 2-DG-treated animals was not due to a difference in the inflammatory response (cytokine secretion) itself but due to impaired tolerance to viral inflammation by interference with the endoplasmatic reticulum stress response, which emphasizes the important role of the metabolic state of an organism in the face of viral insults (Wang et al., 2016). The current study is the first to report that fasting exaggerates the inflammatory response to Poly(I:C) challenge, as reflected by an increase of peripherally secreted cytokines, which may explain the aggravated sickness response to TLR3 stimulation following IF.

### Elevated Cytokine Expression in the Periphery as a Mechanism Behind the Amplified Sickness Behavior in IF Mice

One major route of communication between the peripheral immune system and the brain are circulating cytokines that either directly enter the brain via areas such as the circumventricular organs that have a more permissive blood-brain-barrier or stimulate specific receptors at the peripheral fibers of the vagus nerve, both leading to a transfer of the pro-inflammatory stimulus to the brain (Dantzer et al., 2000; Holzer et al., 2017). In order to assess possible mediators of the IF-induced exacerbation of Poly(I:C)-evoked sickness behavior, both circulating cytokines as well as cytokine mRNA expression in the hypothalamus, a brain region involved in both energy balance and central response to peripheral inflammation (Guijarro et al., 2006), were evaluated.

The overall circulating cytokine levels measured in the AL + Poly(I:C) group were lower than the concentrations found in other studies using the same dose of Poly(I:C) (Cunningham et al., 2007; Weintraub et al., 2014; Murray et al., 2015). Apart from assay type and sensitivity, these differences may be due to the purity of Poly(I:C) (i.e., absence of endotoxin), length of the dsRNA molecule used, time of analysis post-injection, and differences in the experimental animals (e.g., strain, sex, housing) (Kato et al., 2008; Michalovicz and Konat, 2014; Murray et al., 2015; Mayerhofer et al., 2017).

The ability of IF to exaggerate the immune response to peripheral TLR3 stimulation is highlighted by the observed elevation of circulating cytokines. Most strikingly, only mice that were both fasted and injected with Poly(I:C) showed a significant increase in TNF-α, IL-6, and IFN-α levels when compared to vehicle-treated controls. In fact, one key mechanism of the exacerbated sickness response to Poly(I:C) by IF might be the enhanced release of TNF-α, since circulating TNF-α is known to be a crucial mediator in the mirroring of pro-inflammatory signals from the periphery to the brain (Bluthé et al., 2000a; D’Mello et al., 2009). Hence, this cytokine is very likely to contribute to the augmented behavioral sickness response in the IF group. The inability of Poly(I:C) alone to significantly elevate plasma TNF-α might be a factor explaining the absence of an overt sickness behavior in the novel environment of the OF. Furthermore, the enhanced secretion of IL-6 and IFN-α in the Poly(I:C)-treated IF group found in the current study could explain the advanced sickness response. IL-6 is known to be required for the manifestation of behavioral responses to systemic immune stimulation, and IFN-α in concert with IL-6 is thought to additively induce hypoactivity (Bluthé et al., 2000b; Murray et al., 2015).

Additionally, IFN-γ and MCP-1 levels were elevated significantly only in IF mice treated with the dsRNA mimic. Although IFN-γ alone is not likely to induce a strong sickness response, it is believed to play a role in maintaining the expression of other cytokines in chronic stress models (Litteljohn et al., 2010; McCusker and Kelley, 2013). IFN-γ may therefore be another factor of relevance, especially in view of the mild stress that is likely to occur with IF.

The IF-related boost of the overall cytokine response to Poly(I:C) included the anti-inflammatory cytokine IL-10, which is in line with a similar increase in circulating IL-10 levels 4 h following LPS injection to calorie-restricted rats (MacDonald et al., 2014) and might reflect an attempt to limit excessive inflammation. IL-10 is an important immunoregulatory factor as it is known to inhibit the TLR4-mediated induction of pro-inflammatory cytokines such as TNF-α, IL-6, and IL-1β (Fiorentino et al., 1991; Grutz, 2005). The present findings indicate that TLR3 activation triggers a similar mechanism orchestrating the overall immune response.

Moreover, plasma IFN-β was increased to a lesser extent than in other studies following treatment with the same Poly(I:C) dose (Cunningham et al., 2007; Murray et al., 2015). This discrepancy is difficult to disentangle because the size and structure of the dsRNA molecule used are not specified in detail and because female mice were used.

Given the inefficacy of IF to impact the Poly(I:C)-induced cerebral cytokine mRNA expression, our results suggest that the circulating cytokine levels enhanced by IF have a bigger influence on the exacerbated behavioral sickness response to Poly(I:C) as compared to central cytokine expression. In the hypothalamus we quantitated cytokine expression only at the mRNA level for three reasons. First, the assay of cytokine mRNA in the brain enabled us to measure cerebral cytokine expression independently of any cytokines that have entered the brain via the circulation. Second, quantitation of cytokine mRNA reflects the dynamic process of gene transcription (Maier et al., 2009) at the peak of the behavioral response to Poly(I:C). Measurement of cytokine mRNAs in the brain has also been the preferred method to characterize the neuroinflammatory response to LPS (Turrin et al., 2001; Bay-Richter et al., 2011). Third, previous work in the authors’ laboratory has shown that the pattern of cerebral cytokine expression 3 h after LPS treatment is similar at the mRNA and protein level although the LPS response is more sensitively mirrored by mRNA than protein changes (Farzi, 2015).

### CORT Is Elevated in IF Animals, but Does Not Influence the Cytokine Response to Poly(I:C)

The IF regimen markedly enhanced the CORT levels in the plasma of vehicle-treated mice, which is in accordance with other reports in rodents showing that circulating CORT rises both after a brief fasting episode as well as calorie restriction over short or long periods (Wan et al., 2003; Lowette et al., 2014; MacDonald et al., 2014). Although the Poly(I:C)-treated IF group presented with significantly enhanced plasma CORT levels, the IF-related change in glucocorticoid secretion did not influence the pro-inflammatory effect of the dsRNA mimic. By inference, the immune response to TLR3 stimulation seems to be less sensitive to glucocorticoid inhibition than the response to TLR4 stimulation, given that LPS-induced inflammation and sickness are thought to be blunted by calorie restriction through stimulation of CORT secretion (Matsuzaki et al., 2001; MacDonald et al., 2014). Subsequently, the elevated CORT levels caused by metabolic stress due to IF might contribute to a bouquet of effects of the viral mimic in the central nervous system. Of note, IF animals treated with Poly(I:C) presented with lower CORT levels than vehicle-injected IF mice, which suggests that Poly(I:C) might dampen the IF-stimulated HPA axis. A similar observation was made in a study of bacterial infection in which lower CORT levels were found in IF mice after *Salmonella typhimurium* infection than in non-infected mice (Godinez-Victoria et al., 2014). This might reflect a negative feedback mechanism instigated by IF, blunting the release of CORT and its effect on the central nervous system (Dallman et al., 1987; Herman et al., 2012).

### Elevated NPY Levels in the Hypothalamus Do Not Abate Sickness in IF Mice Treated With Poly(I:C)

The orexigenic neuropeptide NPY is expressed throughout the peripheral and central nervous system and thought to protect against behavioral disturbances in response to immune challenge (Ferreira et al., 2011, 2012; Farzi et al., 2015a; Radler et al., 2015). In line with other reports (White and Kershaw, 1990; Chua et al., 1991; Marks et al., 1992; Gotthardt et al., 2016), IF significantly increased NPY expression in the hypothalamus. Unlike immune stimulation by bacteria-derived components (Freund’s adjuvant, LPS), which impacts the cerebral NPY system (Ji et al., 1994; Kim et al., 2007), Poly(I:C) failed to significantly boost hypothalamic NPY expression. It has been proposed that the effect of fasting to attenuate TLR4 (LPS)-mediated microglial activation may indirectly be due to NPY induction in the brain (Radler et al., 2015). In the present study, however, the IF-evoked elevation of NPY expression in the hypothalamus failed to suppress the sickness response to TLR3 stimulation. Whether NPY is indeed unable to blunt Poly(I:C)-induced sickness behavior awaits to be explored by the use of NPY antagonism or deletion.

Nevertheless, NPY may differentially affect TLR3- and TLR4-mediated disturbances of brain function and behavior, as there are some distinct differences in the mechanisms whereby Poly(I:C) and LPS induce immune responses and behavioral changes (Hopwood et al., 2009; Kong et al., 2015; Wang et al., 2016). Thus, i.p. LPS is thought to enter the circulation and subsequently to access various peripheral and central sites of action including the blood-brain-barrier and cells of the circumventricular organs (Lenczowski et al., 1997; Romanovsky et al., 2000). Poly(I:C), on the other hand, is readily degraded by ubiquitous RNases and therefore will only activate TLR3 present at the site of injection (Lenczowski et al., 1997; Romanovsky et al., 2000; Konat, 2016). As a consequence, peripheral LPS and Poly(I:C) take different routes to signal to the brain, which is likely to explain why bacterial and viral immune stimulants act on different brain regions (Wang et al., 2016). Finally, glucose deprivation is known to differentially influence immune stimulation by LPS and Poly(I:C) and has beneficial effects during bacterial, but deleterious consequences during viral, inflammation (Wang et al., 2016).

## Conclusion

Taken together, this study shows that (I) the TLR3 stimulant Poly(I:C), given i.p., induces a sickness response attenuating physical activity and ingestive behavior in a homecage environment 4–6 h post-injection. (II) TLR3 signaling causes peripheral secretion and hypothalamic expression of pro-inflammatory cytokines. (III) IF exacerbates the effects of Poly(I:C) to stimulate peripheral cytokine expression and the sickness response. (IV) This fasting regimen enhances the plasma levels of CORT and the expression of NPY in the hypothalamus, but these molecular reactions do not prevent the immune and behavioral responses to TLR3 stimulation. Collectively, our findings highlight an adverse influence of IF on TLR3-mediated immune stimulation and sickness behavior which is likely mediated by the boost in peripheral cytokine levels.

## Ethics Statement

This study was carried out in accordance with the recommendations of the Directive of the European Parliament and of the Council of 22 September 2010 (2010/63/EU). The protocol was approved by an ethical committee at the Federal Ministry of Science, Research and Economy of the Republic of Austria (BMWF-66010/0102-WF/V/3b/2017). Special care was taken and the experiments were designed in such a way that the suffering and the total number of animals used was minimized.

## Author Contributions

GZ provided the study concept as well as the design, performed all experiments, acquired the data, and wrote the manuscript with supervision of PH. AJ helped with the behavioral experiments. PH, AF, and FR supervised and contributed to the design of the study. All authors read and approved the final manuscript.

## Conflict of Interest Statement

The authors declare that the research was conducted in the absence of any commercial or financial relationships that could be construed as a potential conflict of interest.

## Abbreviations

ACTB: actin beta
AL: *ad libitum*
ANOVA: analysis of variance
CORT: corticosterone
dsRNA: double-stranded RNA
HPA: hypothalamic-pituitary-adrenal
i.p.: intraperitoneal
IF: intermittent fasting
IFN: interferon
IL: interleukin
LPS: lipopolysaccharide
MCP-1: monocyte chemoattractant protein 1
NPY: neuropeptide Y
OF: open field
PAMP: pathogen-associated molecular pattern
Poly(I:C): polyinosinic:polycytidylic acid
PPIL3: peptidylprolyl isomerase like 3
PRR: pattern recognition receptors
TLR: Toll-like receptor
TNF: tumor necrosis factor
VEH: vehicle
